# Improving access to eye care for women and girls: practical steps

**Published:** 2025-03-07

**Authors:** Fiona Lawless, Tiangey Gondoe, Astou Sarr

**Affiliations:** 1 Health Policy Adviser: Sightsavers, UK.; 2 Country Director – Sierra Leone: Sightsavers.; 3 Country Director – Burkina Faso & Côte D’Ivoire: Sightsavers.

**There is much that eye health professionals and managers can do to improve access to eye care for women and girls in their clinics or eye health programmes.**

**Figure:**
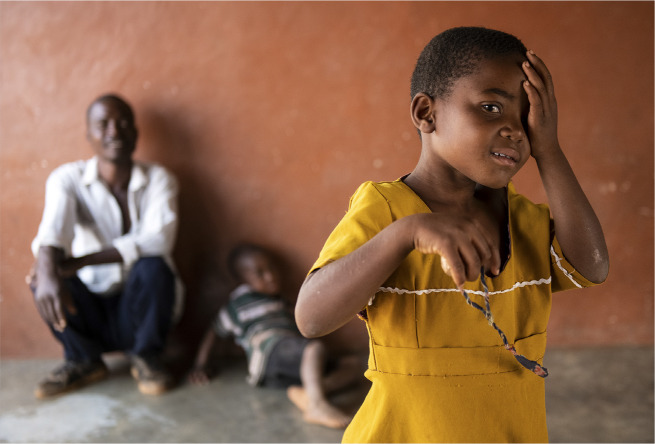
Involve men in campaigns to promote eye care for women and girls. Here, a father looks on while his 8-year-old daughter undergoes vision screening at an eye camp in rural Malawi. malawi

© SIGHTSAVERS/THOKOCHIKONDI CC BY-NC-SA 4.0

Initiatives that make eye care services more affordable and bring them closer to communities can help to overcome several of the barriers faced by women, as described elsewhere in this issue (see page 4). By specifically targeting women and girls, these initiatives can be improved; for example, by working with local women's groups or carrying out eye screening in workplaces dominated by women such as hospitals, health centres, and garment factories.

However, if we are to provide access to eye care for **all** the women and girls who need it, we need to do more. In this article, we look specifically what else health care workers and managers can do in their health facility or eye health programme.

## Step 1. Data collection

You cannot start to solve a problem without knowing it exists – or how big it is. Gathering and analysing data by gender is necessary so that we can see if there are any differences between women and men's (or girls and boys’) access to eye care services, such as cataract surgery or refractive error screening, and how great those differences are.

To find out if women and men are receiving eye care in proportion to their needs, calculate the percentage of women and men accessing eye services at your facility or in your eye programme, and compare this to what is known about the percentage of women and men with eye care needs in the community.

To carry out a very broad gender analysis across all age groups and eye conditions, you can take the following steps:

1. Analyse the eye health department or eye programme records by gender: divide the total number of female patients by the total number of patients of all genders, and multiply this by 100. This gives you the overall percentage of female patients accessing the service.
2. Compare this to the percentage of women and girls in the community or country who are known to have eye conditions. You can use local rapid assessment of avoidable blindness (RAAB) data, if available for your country (available at www.raab.world). Or, if no national or local data is available, we know from global data that approximately 55% of those who are blind or who have vision impairment are female.

If you have access to eye service/facility and national data for different eye conditions and/or age groups you can make similar comparisons to gain a more detailed understanding of any gender differences (disparities) in access, e.g. amongst people over the age of 60 years coming for cataract surgery, or in school-age children receiving refractive error screening. It may also be useful to gather data on other characteristics, such as disability status, or urban vs rural location, in order to identify which women and girls face additional barriers to accessing services.

### TIP: Keep on collecting data

Data collection isn't a one-time activity. As you work to improve women and girls’ access to eye services, continuously monitor the gender of people using the service. This will allow you to track the success of each new initiative you try. This feedback is important as it tells you how well you are doing, and may highlight new areas that need improvement.

## Step 2. Consult with key groups

The data analysis in Step 1 will show you whether disparities exist, and where they are. To understand what is preventing women and girls from accessing eye health services, and how to overcome these barriers, you will need to speak with them. You can do this either individually, through conversations, or collectively, through focus group discussions – for example, with women who are members of local community groups. You could also speak with groups such as community leaders and community health workers who work closely with women. Ask these groups why they think women are not accessing eye health services, and what they think will make things easier or better. Please note that this should be done with each group separately, and where possible with women and men separately too. Women may feel scared or unwilling to discuss sensitive issues when male community members are present.

The 2012 *Community Eye Health Journal* issue ‘Putting patients at the centre of eye services’ includes practical suggestions on finding out what patients think. Visit https://cehjournal.org/38/volume/25/issue/78 to read more.

### TIP: Consider all women and girls

When consulting with different groups, consider women and girls in all their diversity. Women and girls with different characteristics, such as age, disability, income levels, or membership of indigenous or specific ethnic groups, will face multiple, compounding barriers that hinder their access. For example, women who live in poverty, women with disabilities, and indigenous women all have additional barriers to overcome in accessing health care. Understandin and addressing these ‘intersectional’ barriers is only possible through consultation with women and girls from all of these groups.

## Step 3: Finding solutions

Specific barriers will need specific solutions, which should be developed in conjunction with the communities involved. Women – and women leaders – must be at the heart of these discussions, but we also need to involve cultural and religious leaders to ensure that actions taken are appropriate and acceptable to everyone in the community. This step could be taken in conjunction with the above consultation process.

**Case study: Ethiopia**

**Figure 1:**
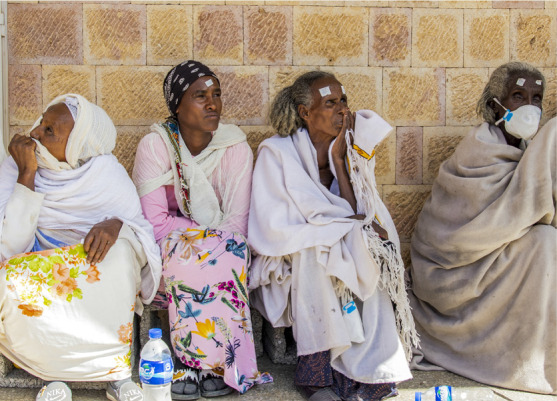
Establishing a separate waiting line for women increased their access to eye care.

One example of tailored programming from Ethiopia showed that establishing two waiting lines during outreach programmes – one for women and one for men – increased access to eye health for women. This is because, due to women's household and caring responsibilities, they may not be able to show up early to stand in long lines and could miss out on a consultation or operation otherwise (Figure 1).

© GENAYE ESHETU CC BY-NC-SA 4.0

**Case study: Malawi**

Health care workers at eight district hospitals received gender training in 2020 and 2021, which helped them to better understand the different barriers that women face in trying to access eye care. For example, they realised that the distance people needed to travel, and the cost of this, was keeping women from coming for cataract surgery, Opthalmologist Dr Moira Chinthambi explains: “The team now sends vehicles to the remotest of areas to look for women and girls and make sure they're included. Sometimes people would have to walk to go and look for patients and pick them up and bring them to the nearest health facility. That also helped with the economic challenges, because now there's no need for transport money.”

**Figure:**
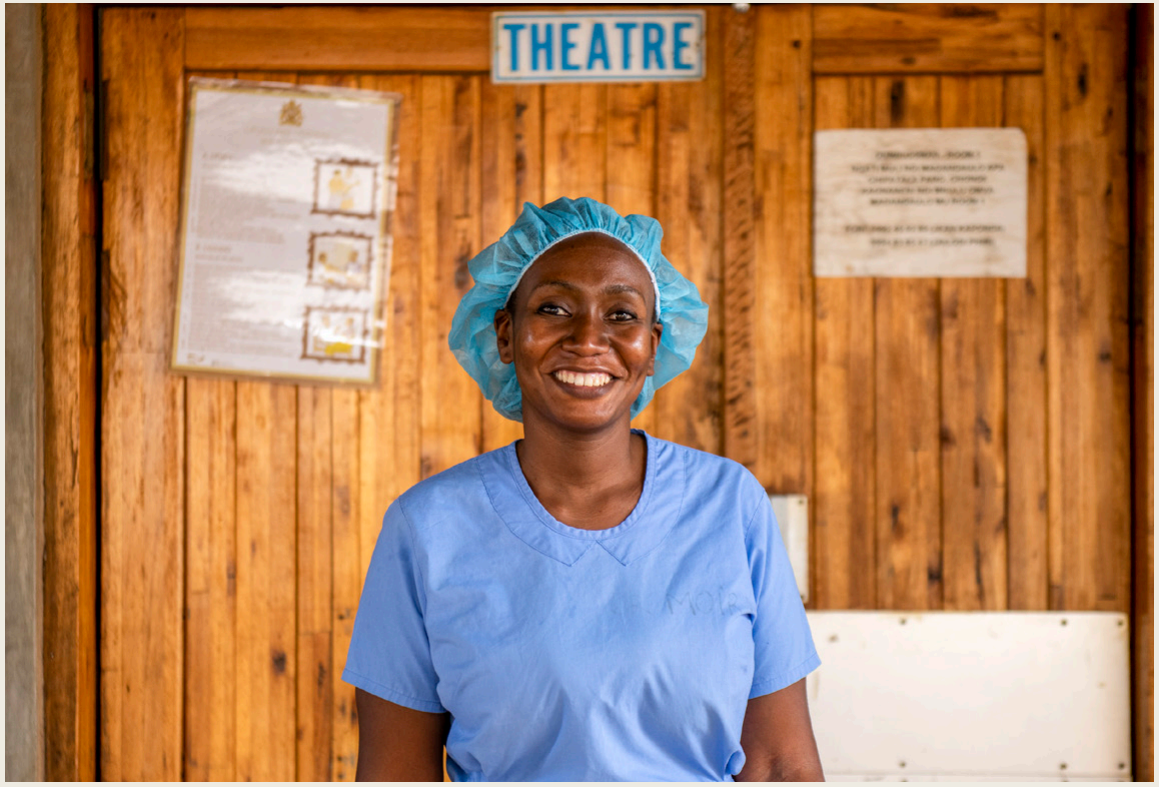
Dr Moira Chinthambi. malawi

© LIGHT FOR THE WORLD CC BY-NC-SA 4.0

Ensure that women's voices shape the solutions. Engaging them in identifying challenges and co-designing practical, culturally appropriate strategies will lead to more effective and sustainable change. These insights can then be brought to eye care management, not just as problems, but as collaboratively developed solutions that reflect the real needs and priorities of the women affected.

“Engaging women in identifying challenges and co-designing practical, culturally appropriate strategies will lead to more effective and sustainable change.”

Another common challenge is when women do not feel comfortable seeing a male professional. This could be overcome with women-specific clinics or specific scheduling of female eye care practitioners. In Sierra Leone, for example, some women refused to be treated by male professionals due to their religious beliefs and because they did not feel comfortable around men. Setting up specific clinics for women, with women eye care practitioners, helped them to feel comfortable to seek care.  

Something as simple as allowing women to bring their children to the eye clinic can also help, as women are often the default child care provider and would otherwise need to find someone to look after their children.

### TIP: Remember that eye care is part of health care

Reach out to other health professionals to explore how you can work together to improve access for women. For example, you can agree to offer eye health services near and/or on the same days as other health services commonly used by women, such as maternal and child health clinics. This will reduce the number of trips and the associated costs.

## Step 4. Educate health workers

Many of your colleagues may not grasp the depth of inequities in health care or how these can affect access to health care. You can:

1. Host an information session highlighting these inequities, share real world examples, and brainstorm actionable solutions.
2. Conduct training to create awareness of traditional gender roles and expectations. This can include training on gender biases and how we, as health care workers, can challenge our own assumptions or biases, such as assuming that children need to be looked after by women, when in reality men are equally capable of doing so.
3. Present ideas to management or co-create solutions to the barriers you have identified.

## Step 5. Address cultural and gender expectations in the community

Ingrained cultural and gender expectations can be more complex to address, but eye care professionals are trusted members of the community and can speak with community and religious leaders about the need for women to have eye care. It can be helpful to work together with other health professionals, e.g. maternal and child health workers.

**Figure 2:**
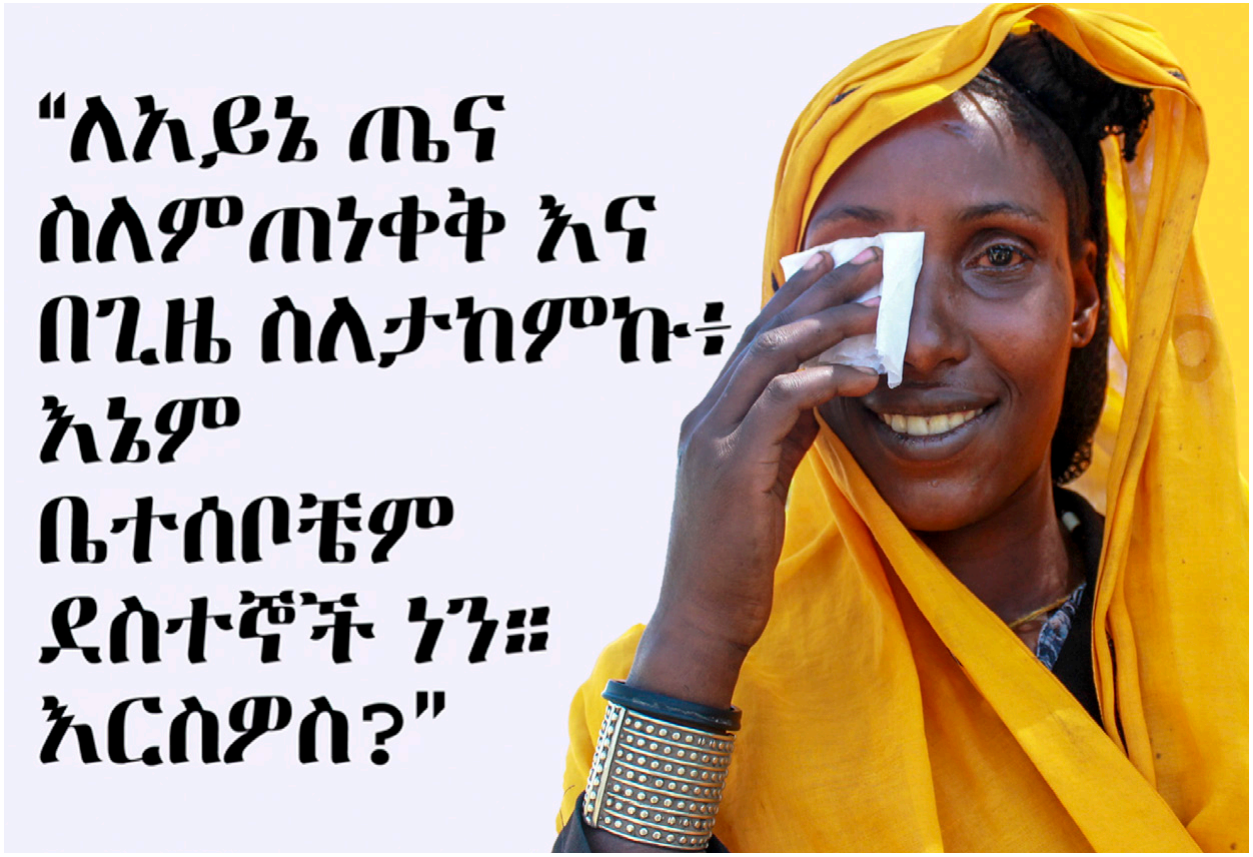
Billboards and posters designed to encourage women to prioritise their own eye health. The text reads: ‘Keep your eyes healthy, keep your family healthier”. ethiopia

Simple health promotion messages can be very useful. One of the barriers highlighted in this issue is that women may prioritise their family's eye care needs over their own. Light for the World addressed this in Ethiopa by creating posters and billboards encouraging women to prioritise their own eye health (Figure 2) as part of a wider project supported by the Austrian Ministry of Social Affairs, Health, and Consumer Protection.

© LIGHT FOR THE WORLD CC BY-NC-SA 4.0

### TIP: include men

Messages aimed at men – about the importance of women's access to eye care, and the benefits of this – are needed to challenge patriarchal norms in some societies. This can include messages about the need to provide money and other support for female family members to access eye care services, particularly when they have to travel to secondary facilities after being identified at a screening or outreach programme, and messages about the need for men to perform domestic duties and take over caring responsibilities so that women can travel to receive eye care and have time to recover after surgery.

**Case study: Involving men to improve access for women**

In a Sightsavers- and UK Aid-funded programme in Tanzania, the team improved access to eye care for women by working directly with men to challenge attitudes and change their behaviour.

Programme manager Edwin Maleko explained: “Around 56% of the people who benefited from the programme are women, but in terms of cataract surgery, the number in our previous project was just 36%. Why? Because if a woman accepts cataract surgery, she may need around two weeks to recover. Who will take care of the children? That is the challenge. To tackle it, we needed to challenge community attitudes and behaviour, so that men can be more responsible in taking care of their children and doing domestic work.”

The team designed a set of messages aimed at making men more supportive of their spouses attending health care services and clinics (known as a **sensitisation approach**). The team also **engaged with religious leaders** and asked them to encourage men to support women.

In addition, the team worked in partnership with the Tanzania Gender Network to provide training, develop training manuals, and liaise with social welfare officers working at district and regional levels.

“While there is still work to do in this area, it was encouraging to see that during the programme, the number of women who accepted cataract surgery is now 46%. We can see the benefit of all these efforts.”

**Figure:**
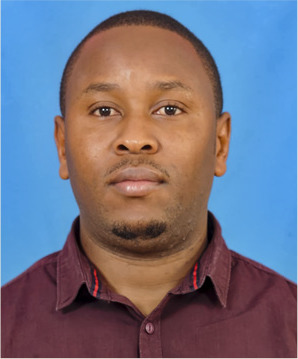
Edwin Maleko tanzania
